# Successful Treatment with Brigatinib after Alectinib-Induced Hemolytic Anemia in Patients with Metastatic Lung Adenocarcinoma—A Case Series

**DOI:** 10.3390/curroncol30010041

**Published:** 2022-12-30

**Authors:** Rola El Sayed, Mustapha Tehfe, Normand Blais

**Affiliations:** Centre Hospitalier de l’Université de Montréal, Montréal, QC H2X 0A9, Canada

**Keywords:** non-small-cell lung cancer, alectinib, oxidative stress, hemolytic anemia

## Abstract

Alectinib is a second-generation anaplastic lymphoma kinase (ALK) inhibitor used in the treatment of advanced ALK-rearrangement positive non-small-cell lung cancer (NSCLC). Many tolerable adverse events were reported with the use of Alectinib; nevertheless, hemolytic anemia was not mentioned in the safety analysis. In this case, series, we report four cases of Alectinib-induced oxidative hemolytic anemia and discuss different etiologic hypotheses on the underlying mechanism of such overlooked adverse event of the drug. Furthermore, we draw attention to the successful treatment with Brigatinib, an alternative second-generation ALK-inhibitor without recurrence of hemolytic anemia in three of our four cases, suggesting a probable class effect.

## 1. Introduction 

The incorporation of molecular assays into the diagnostic work-up of non-small-cell lung cancer (NSCLC) has tremendously changed the treatment paradigm of lung malignancy [1]. Targeted treatment has led to outstanding improvement in disease control with a shift in the prognostic horizon of patients with advanced lung cancer [2,3]. Five-year survival rates of up to 60% can be achieved with some targeted agents [4,5], and advances in precision medicine continue to discover more potent domain-targeted tyrosine kinase inhibitors [6]. Nevertheless, these orally bioavailable convenient systemic therapy options are not devoid of adverse events [7].

In this case review, we will be discussing a particular adverse event noted with the use of one of the new tyrosine kinase inhibitors specifically targeting ALK-rearrangements. The latter can be found in up to 5% of all cases of NSCLC [8], more frequently in never- or light-smokers [9]. Multiple ALK-inhibitors exist including: first generation Crizotinib, second generation Ceritinib, Brigatinib, Alectinib, and third generation Lorlatinib [10], Entrectinib [11], and Ensartinib [12]. Based on efficacy and superiority data derived from randomized clinical trials: ALEX [13], J-ALEX [14], ALESIA [15] and ALUR [16], Alectinib became a preferred first-line treatment of ALK-positive NSCLC [17]. Alectinib-related grade 3 adverse events were reported in up to 52% of patients in ALEX trial. They included: anemia, liver enzyme abnormalities, creatinine phosphokinase elevation, increased risk of pneumonia and rash. Dose reduction was required in up to 20% of cases, and treatment interruption in up to 26%. At the cut off data date of the 29th of November 2019, the percentage of fatal adverse events was estimated between 0 and 4.6% depending on the population studied [5,14,15]. 

Furthermore, anemia was reported in up to 22% of patients on Alectinib, with only 5% of patients developing grade 3 or more [5]. All cases of anemia were considered directly drug-related with no mention of Alectinib-induced hemolysis in the safety analysis of the clinical trials. However, few case reports have been published discussing Alectinib-induced alteration in erythrocyte morphology and membrane structure [18,19], specifically Alectinib-induced acanthocytosis, as well as sphero-acanthocytosis [20]. In addition, Alectinib was shown to interfere with eryptosis. It caused cell membrane scrambling following energy depletion, leading to a hyper-osmotic shock that accumulates defective erythrocytes and increases the risk of hemolysis [21]. No report was found as to whether alternative ALK-inhibitors can be attempted in such cases.

Accordingly, we will be presenting our own experience of Alectinib-induced hemolytic anemia in 4 patients diagnosed to have stage IV ALK-positive NSCLC treated at our center. Of note, we portray the successful retreatment of 3 of these patients with an alternative second-generation ALK inhibitor Brigatinib without recurrence of hemolysis. Further on, we will be tackling a few hypotheses as well on the mechanisms of development of such hemolytic anemia which seems to be a rather class effect among ALK inhibitors.

Informed consent was taken from patients or their closest of kin (in case of death).

## 2. Case Reports

### 2.1. Patient 1

**Figure 1:**
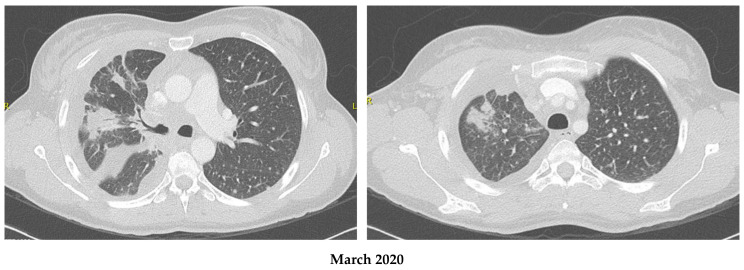

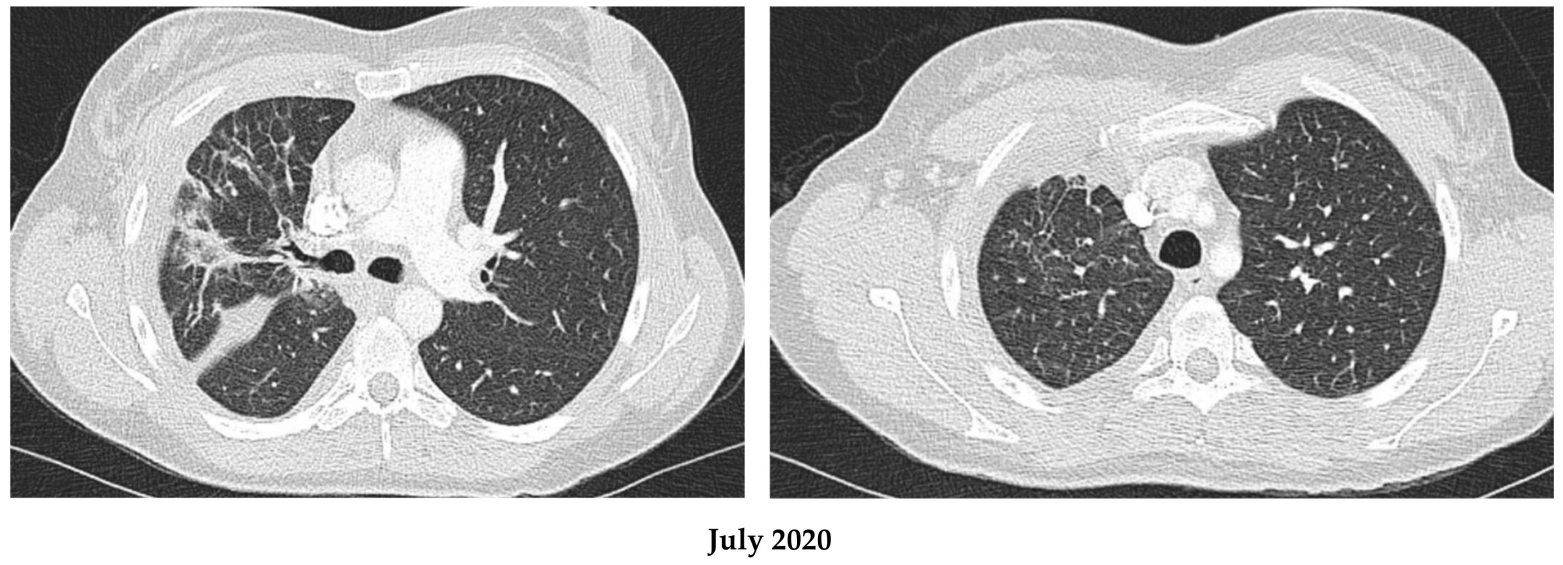
Disease response on Alectinib in patient 1. (Comparison between March and July 2020; 3 months of treatment).

A 45-year-old Caucasian female, smoker, presented initially for dyspnea and chest pain in March 2020. Work-up revealed an advanced right lung malignancy with involvement of the pleura, liver, and bones. Pathology showed a primary pulmonary adenocarcinoma, ALK-rearrangement positive, a programmed cell death ligand 1 (PD-L1) of >50%, and no other molecular mutation was found. The patient was diagnosed to have a stage IV, cT3N3M1c ALK-positive adenocarcinoma, and was started on first line Alectinib at a dose of 600 mg twice daily in April 2020.The patient had an excellent response both clinically and radiologically. (Figure 1).

In July 2020, a perturbation in liver enzymes was noted with a progressive rise in bilirubin reaching 65 μmol/L [7–23 μmol/L] (indirect bilirubin of 31 μmol/L), aspartate aminotransferase (AST) reaching 1309 U/L [13–39 U/L], and alanine aminotransferase (ALT) reaching 1330 U/L [8–31 U/L]. On the other hand, alkaline phosphatase (ALP) showed minor alteration at 144 U/L [36–110 U/L], suggesting a hepatic cytolytic pattern. Clinically, the patient had started complaining of lack of energy and anorexia, but no hospitalization was required. Liver virology testing for hepatitis B, C and HIV turned out negative. Liver-directed ultrasonography showed a decrease in liver metastases, mild steatosis, and no parenchymal dysmorphism. Shortly after, a hemoglobin (Hb) drop was reported from 121 to 112 g/L, with a maintained mean corpuscular volume (MCV) of 92–94 fL, normal platelets (Plt) in the range of 250–300 × 10^9^/L, yet an elevated lactate dehydrogenase (LDH) reaching 575 U/L, elevated reticulocyte count (retic) reaching 105.8 × 10^9^/L, an indirect bilirubin of 34.2 μmol/L, a haptoglobin of <0.3 g/L, and a negative direct and indirect Coombs test. Moreover, the peripheral smear showed multiple acanthocytes, echinocytes and spherocytes, with only few schistocytes.

Auto immune and erythrocyte enzymatic panels were performed. Auto-immune work up including anti-smooth muscle antibodies, anti-mitochondrial antibodies, anti-nuclear antibodies, showed only a non-specific positive ANA (both granular and nucleolar at a titer of 1/160). Total serum IgG levels were found to be elevated (13.91 g/L) in favor of a chronic inflammatory process. The erythrocyte enzymatic panel including G6PD, glucose phosphate isomerase, pyruvate kinase, hexokinase, and glutathione reductase was normal. 

The patient was diagnosed with grade 3 liver toxicity as well as Alectinib-induced Coombs negative hemolytic anemia. Alectinib was stopped as of late august 2020, with a gradual normalization of all laboratory values including: aminotransferases, bilirubin, LDH, and Hb. The patient was allowed a 4-week recovery period before 2^nd^ line Brigatinib was initiated as of October 2020. No further similar lab perturbations were noted.

### 2.2. Patient 2

**Figure 2:**
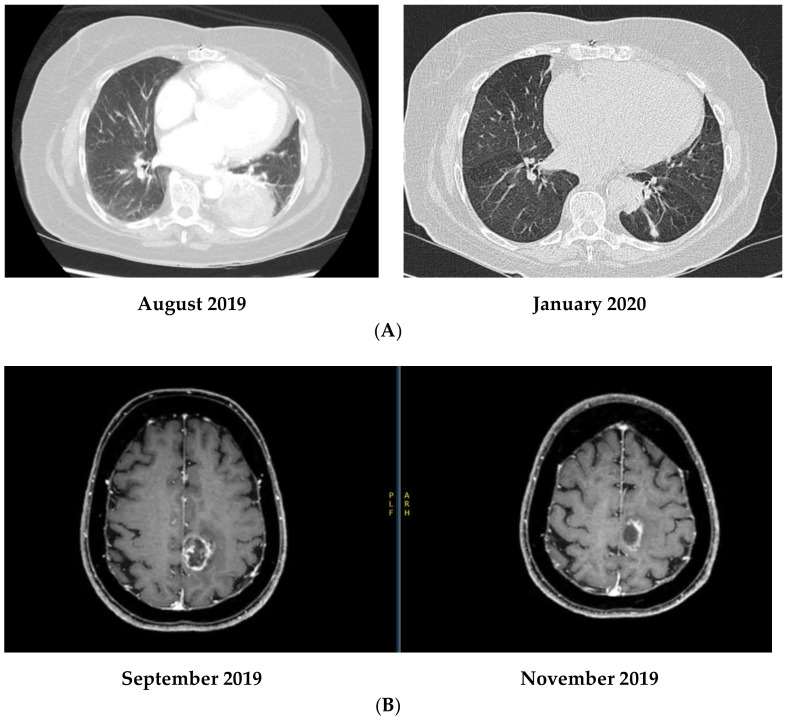
(**A**) Disease response on Alectinib in patient 2. (Comparison between August 2019 and January 2020). (**B**) Variation of brain lesion in patient 2 on Alectinib within less than one month on treatment.

An 80-year-old middle eastern female, smoker, with multiple comorbidities including a baseline normocytic anemia of chronic disease with a Hb of 106 g/L, was undergoing aortic valve replacement surgery work-up when a large left lung lesion with contralateral mediastinal lymph nodes, and a unique brain metastatic lesion were discovered. Endoscopic biopsy was positive for lung adenocarcinoma, ALK positive, PDL-1 > 50%, and negative for all other known mutations by next generation sequencing. She was diagnosed with stage IV, ALK-positive lung adenocarcinoma, cT4N3M1b, and was started on Alectinib 600 mg BID as of October 2019. In early November 2019, there was a significant radiologic response (Figure 2); yet the patient was hospitalized for rapid atrial fibrillation resulting in cardiac insufficiency related to her severe aortic stenosis and of a newly worsening anemia, where Hb dropped to 67 g/L, with an MCV of 83fL [80–100 fL] (normocytic). LDH was increased up to 361 U/L [104–205 U/L], and reticulocyte count increased up to 143 × 10^9^/L. There was a slow rise of indirect bilirubin up to 25 μmol/L in addition to a drop in haptoglobin to <0.3 g/L. Coombs direct and indirect testing were negative. A G6PD panel was also performed and turned out negative. Iron testing showed a serum iron level of 6.6 µmol/L, iron/transferrin saturation 11%, and a ferritin of 54 µg/L. All other lab tests were within the normal range including liver enzymes. The peripheral smear showed anisocytosis, multiple acanthocytes, as well as echinocytes. No hepatosplenomegaly was noted on physical examination.

She received blood transfusions, IV iron and erythropoietin in addition to optimization of her cardiac function. Upper and lower endoscopies to rule out blood loss were performed and turned out negative. Alectinib dose was maintained at the time despite a minor interruption early in the hospitalization (1 week, continued at same initial dose).

With a persistent hemolytic picture and a Hb in the 80–90 g/L range, Alectinib dose was reduced to 450 mg BID in March 2020. Thereafter, Alectinib was stopped in early April 2020 with a Hb rising to 133 g/L on follow-up testing. At this point, a rechallenge of Alectinib was attempted in late June 2020, after trans-catheter aortic valve replacement (TAVI) with a biologic valve in early June 2020. Unfortunately, Hb dropped again with the same hemolytic picture leading to permanent discontinuation of Alectinib in July 2020. Consequently, Brigatinib was initiated as of August 2020. All laboratory tests improved rapidly with Hb back to 133 g/L, bilirubin within normal range, and haptoglobin of 2.22 g/L (Table 1), all confirming Alectinib-related hemolytic anemia.

### 2.3. Patient 3

**Figure 3:**
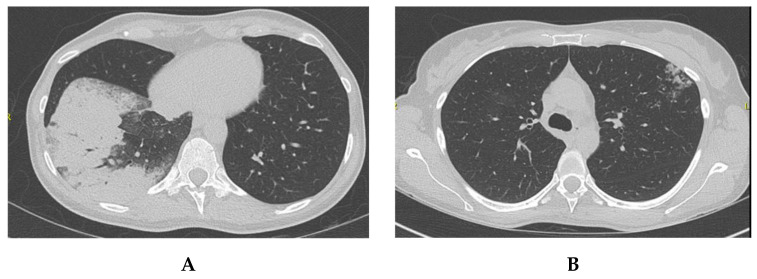

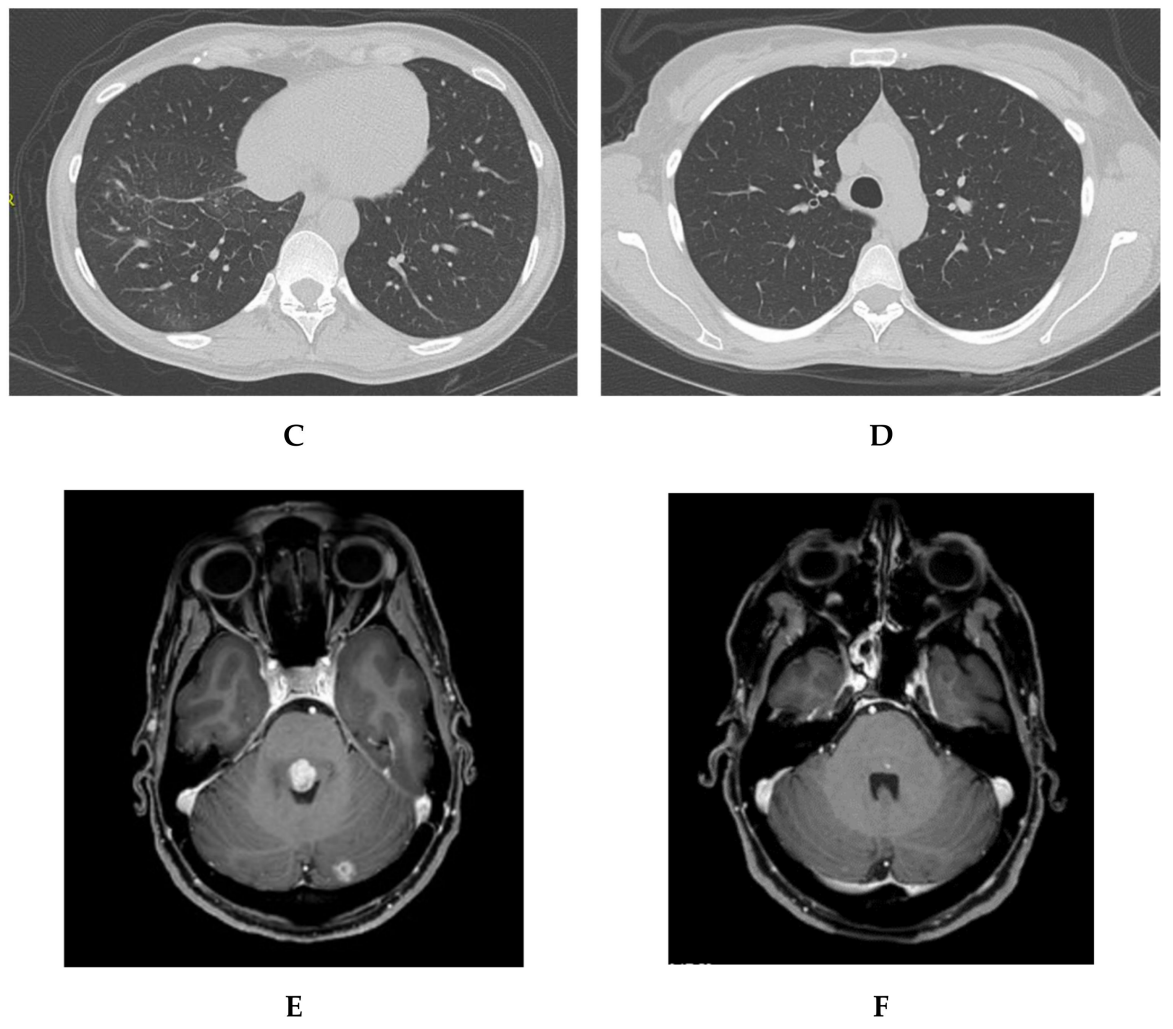
Disease Response on Alectinib in patient 3. Pictures (**A**,**B**): November 2019. Pictures (**C**,**D**): same localization as A and B, respectively, in January 2020. Pictures (**E**,**F**): Significant response in brain lesions on Alectinib in patient 3. (**E**) November 2019. (**F**) December 2019.

A 48-year-old Caucasian female patient, non-smoker, very active, with no previous medical condition, but a strong family history for malignancy, presented with dry cough, difficulty breathing, as well as dizziness and occasional loss of equilibrium in late October 2019. Work-up lead to the diagnosis of a metastatic left lower lobe lung adenocarcinoma with 3 brain lesions, cT2N2M1c, ALK rearrangement positive, all other mutations negative, and a PD-L1 at 70%. She was started on Alectinib 600 mg BID as of the 1st of November 2019, with a remarkable clinical and radiologic response after only a few days on treatment (Figure 3).

In late February 2020, an increase in indirect bilirubin was noted reaching 26.6 μmol/L (total bil 39 μmol/L), accompanied with an elevated LDH of 285 U/L, an increased retic count of 110 × 10^9^/L, and a low haptoglobin of less than 0.08 g/L. On the other hand, Hb was at 114 g/L, and all other liver enzymes were normal. Ferritin was at 72 µg/L, and vitamin B12 as well as folate were normal. A peripheral smear was performed, and it showed acanthocytes, echinocytes, spherocytes and anisocytosis. Coombs direct and indirect testing were also performed and were negative.

Alectinib-related hemolytic process was suspected and Alectinib was held for 2 weeks. Some improvement in laboratory tests was noted with normalization of Hb reaching 127 g/L, bilirubin of 19 μmol/L and an LDH of 197 U/L. Alectinib was resumed for 3 weeks. Nevertheless, the hemolytic process recurred in a more pronounced manner with scleral icterus, dark urine as well as significant fatigue. Blood tests showed a mildly decreased Hb of 119 g/L, bilirubin of 37 μmol/L, and an LDH of 243, with a haptoglobin of <0.3 g/L. Blood film inspection once more showed acanthocytes as well as echinocytes and spherocytes. 

Alectinib was finally stopped and changed for Brigatinib in June 2020, with the resolution of hemolysis, jaundice, and return of all laboratory tests to normal range, suggesting a direct causality of Alectinib. 

### 2.4. Patient 4

**Figure 4:**
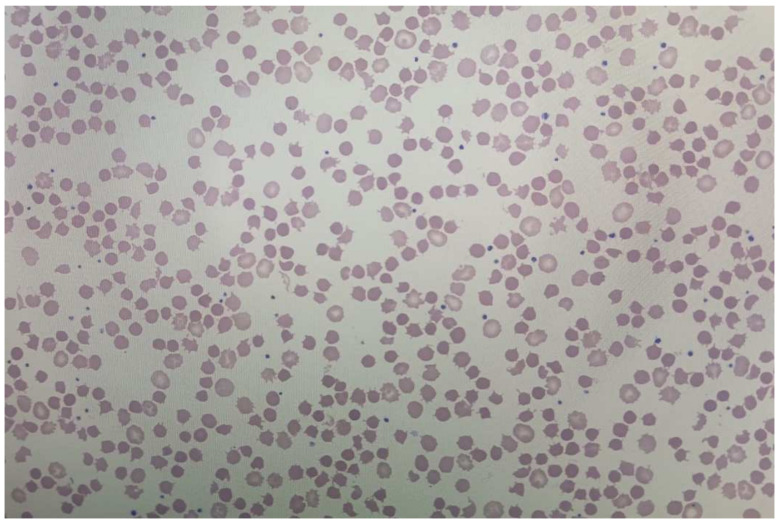
Peripheral Smear of patient 4 showing evidence of hemolytic anemia (presence of spherocytes, bite cells, echinocytes and acanthocytes).

A 69-year-old female patient of Chinese origin, never smoker, was diagnosed with stage IV, ALK positive, PD-L1 negative, and all other mutation negative lung adenocarcinoma after presenting with progressively worsening dyspnea. The patient was started on Alectinib 600 mg twice daily as of February 2022. Early March 2022, the patient was noted to have anemia with a drop of Hb from 126 g/L to 101 g/L, with anMCV of 80 fL. Further testing of iron, vitamin B12, folate and thyroid stimulating hormone was normal. Biochemistry showed indirect hyperbilirubinemia reaching 21 μmol/L (direct bil was 6 μmol/L), an elevated LDH at 298 U/L, a decreased haptoglobin at less than 0.3 g/L, and an elevated retic count at 230 × 10^9^/L. Coombs’ testing was negative. All the following criteria favor an ongoing hemolytic process. Peripheral smear (Figure 4) proved hemolysis, and given no other changes in clinical condition, and the absence of any other new medication introduced, the patient was considered to have Alectinib-induced hemolytic anemia. However, considering the asymptomatic presentation of hemolysis with a Hb that remained stable around 100 g/L, and good response on treatment, no changes to Alectinib dosing were performed. The patient is still on the same dosing of Alectinib with a stable Hb of around 100 g/L, persistent mild chronic hemolysis, and no symptoms, under close observation.

## 3. Discussion

Hemolysis is the accelerated destruction of red blood cells (RBC) leading to responsive increased bone marrow erythropoiesis manifested by reticulocytosis. Clinically, hemolysis is coupled with variable degrees of pallor, fatigue, dyspnea, and jaundice. Biochemically, hemolysis is manifested by a decreased hemoglobin, increased reticulocyte count, in addition to indirect hyper-bilirubinemia, elevated lactate dehydrogenase and a decreased haptoglobin level. In general, hemolysis is classified according to the inheritance pattern: inherited versus (vs.) acquired; site of RBC destruction: intra-vascular vs. extra-vascular; and origin of RBC damage: intrinsic vs. extrinsic hemolysis. In auto-immune hemolytic anemia (AIHA), RBC destruction is mediated by auto-antibodies. On the other hand, non-immune causes of hemolytic anemia (HA) can be variable including abnormal RBC membrane, and abnormal RBC enzymes affecting cellular deformability and shape, or fragmentation [22].

Furthermore, drug-induced hemolytic anemia (DI-HA) is a rare disease, with an annual incidence of about 1/10^6^ [23]. Mechanisms of action involved in DI-HA have been classified into 4 categories: hapten-drug adsorption, ternary or immune-complex related and production of true autoantibodies, as well as non-immunologic deposition of proteins on RBCs accelerating cytoplasmic clearing [23,24,25]. In hapten-drug adsorption, drugs firmly attach to proteins on RBC membrane. HA usually takes place due to IgG class antibodies against drug epitopes leading to eventual elimination of RBCs coated with the drug plus anti-drug antibodies in the spleen. On the other hand, immune-related mechanisms include either complement-related direct cell destruction after binding of drug-RBC antibody, or via the development of autoantibodies. In all cases, antibodies are found, and Coombs testing is usually positive [22]. Finally, there have been reported cases of RBC acanthosis and sphero-acanthocytosis possibly related to RBC oxidative stress due to drugs such as Alectinib. The mechanism discussed included Hb auto-oxidation, energy depletion, RBC deformability, and eventually hemolysis [18,19,20,21,26].

Herein we report the cases of 4 patients diagnosed with ALK-positive metastatic lung adenocarcinoma, who developed enzymatic liver disturbances as well as Coombs negative hemolytic anemia considered to be related to Alectinib. In all 4 of our patients, we noted normocytic anemia, indirect hyperbilirubinemia, increased LDH, decreased haptoglobin and an elevated reticulocyte count shortly after initiation of Alectinib (Table 2). No other new medications were introduced. All 4 cases had a negative direct and indirect antiglobulin testing (Coombs). All patients had normal levels of iron, vitamin B12 and folate. No other reasons for hemolysis were found with negative enzymatic panel, and no clinical suspicion of paroxysmal nocturnal hemoglobinuria (PNH) (despite absence of flow cytometric testing). Moreover, in all 4 patients, the RBC membrane was altered with presence of acanthocytes, echinocytes and spherocytes. 

On the other hand, the presence of autoantibodies anti-ANA, in addition to the increase in the total IgG in the first case may have implied the auto-antibody mechanism of DI-AIHA [27], which can be explained by the presumed interference of tyrosine kinase activity with the humoral immune response [28,29]. However, anti-ANA antibodies are a random normal occurrence in up to 40% of the normal population [30], and the increase in IgG is non-specific, and could merely be related to the general inflammatory condition [31]. In addition, the absence of Coombs antibodies makes the hypothesis of autoimmunity less likely, despite the rare presence of a Coombs negative AIHA. The presence of RBC deformation is rather suggestive of possible oxidative stress affecting the cytoskeleton of RBCs and leading to their destruction [32]. Even though the acanthocytosis seen on the peripheral smear of all patients may be partly attributed to the concomitant liver dysfunction due to Alectinib, the echinocytosis as well as the noted spherocytosis are most likely related to Alectinib-related erythrocyte cell membrane deformability, as previously suggested in the literature [18,21]. One could argue for the possibility of PNH in Coombs negative HA. Nevertheless, the temporal correlation of occurrence of anemia with the initiation of Alectinib, its resolution, as well as the normalization of hemolytic parameters after the discontinuation of Alectinib, and re-occurrence upon resumption confirms the direct correlation with the drug with no other likely etiologies. Furthermore, when Alectinib was replaced by Brigatinib in patients 1–3, no hemolysis recurred. 

An interesting observation was that patient one, two and three were noted to have elevated PD-L1 levels. Oncogene-driven tumors including ALK-rearranged NSCLC are known to have an immune-tolerant tumor microenvironment with decreased cytotoxic T-cells and increased regulatory T cells and myeloid-derived suppressor cells. It has been hypothesized that the interaction of PD-L1 status and ALK inhibition may influence response rates, and perhaps adverse events via immune interplay [33,34,35]. However, real-world data showed no correlation, and our fourth patient had a negative PD-L1 yet developed the same pattern of HA although not as severe, and not requiring drug interruption nor discontinuation. Further observation of a larger number of ALK-positive NSCLC patients needs to be performed before any conclusions can be drawn. Moreover, ethnicity did not seem to affect presentation.

Different theories exist on the underlying mechanisms of Alectinib-induced hemolytic anemia. For instance, ALK inhibitors affect pathways involved in RBC vesiculation such as G protein-coupled receptor (GPCR) signaling, the phosphoinositide 3-kinase (PI3K)–Akt (protein kinase B) pathway, the Jak-STAT (Janus kinase–signal transducer, and activator of transcription) pathway, and the Raf–MEK (mitogen-activated protein kinase)–ERK (extracellular signal-regulated kinase) pathway [36]. More so, ERK1 pathway alteration may induce a splenic stress erythropoiesis phenotype, that may as well be involved with RBC vulnerability [37]. In addition, ALK-inhibitors could interfere with EPO signaling; thereby aborting normal RBC differentiation and leading to dysregulation in band 3, and band 4.1 synthesis, 2 proteins that are considered vital for RBC stability [38]. Additionally, different tyrosine kinase inhibitors can be responsible for endoplasmic reticular stress, mitochondrial dysfunction and production of reactive superoxides leading to oxidative stress [39]. No studies have been reported regarding Alectinib- induced nitrosative stress that might be another mechanism of hemolysis. From a simpler perspective, ALK inhibitors are also associated with altered liver enzymes, which are possible indicators of liver damage, that is usually coupled by lipid accumulation on RBC membranes and significant membrane destabilization [5], in addition to the possibility of increased metabolite concentration and thereby increased risk of adverse events.

In our four cases, drug interruption or discontinuation seems to have resolved the issue without any need for further management. However, when re-challenged with brigatinib, an alternative ALK-inhibitor, none of our first 3 patients had recurrence of HA suggesting possibly a class effect that needs to be further investigated. For example, in two analyses comparing brigatinib to alectinib, structural features of brigatinib such as the presence of the di-methyl oxide group among other features were presumed to make brigatinib probably more specific for ALK with a different adverse event profile [40,41].

## 4. Conclusions

As a conclusion, the recognition of Alectinib-induced HA highlights the need to be vigilant for this under-recognized adverse event. A recent report in the American Journal of Hematology reviewed 31 cases of Alectinib-related hemolytic anemia with 32% probable causality versus 68% possible causality. All cases had negative antiglobulin testing with a temporal relation concerning the de-challenge and rechallenge of Alectinib. The review concluded the presence of confirmed correlation with Alectinib exposure which seems mostly of non-immune nature, consistent with our findings [42]. The key identifying features to identify this adverse event is a rise in indirect bilirubin, decreasing haptoglobin and significant changes on blood film inspection. Such an adverse event may be in core a resultant of variable factors including the mechanism of action of ALK inhibitors; however, given the successful retreatment with brigatinib without recurrence of hemolysis signifies a class-effect that warrants further investigation. 

## Figures and Tables

**Table 1:** Variations in hematologic profile in patient 2 with initiation, interruption, rechallenge and discontinuation of Alectinib.

Date	Alectinib	Hb (g/L)	MCV (fL)	PLT (×10^9^/L)	LDH (U/L)	Bil T (μmol/L)	Bil I (μmol/L)	Haptoglobin (g/L)	Retic (×10^9^/L)
2019–10 (early)	(baseline)	106	84	322	Not done	16	Not done	Not done	Not done
2019–10 (late)	600 mg BID	67	83	181	361	21	Not done	1.57	124
2019–11	600 mg BID	80	91	247	363	26	16.7	0.37	143
2019–12	600 mg BID	101	99	365	371	31	21	<0.3	118
2020–01	600 mg BID	89	99	308	306	32	20.6	<0.3	134
2020–03 (late)	450 mg BID	95	99	321	325	34	22.5	Not done	Not done
2020–04	Stopped	128	98	310	388	52	25	0.38	305
2020–06	Stopped	133	98	350	378	24	13.2	1.23	Not done
2020–07	450 mg BID	83	98	381	401	33	14.3	0.37	265
2020–08	Stopped	123	100	265	302	13	Not done	0.99	49.8
2020–09	Replaced by Brigatinib	133	94.7	207	389	5	Not done	2.22	22.7

Note: Script in red indicates abnormal variable values in correlation with Alectinib interruption or discontinuation.

**Table 2:** Summarized patients’ laboratory data upon diagnosis of hemolytic anemia on Alectinib.

	Hb (g/L)	MCV (fL)	Plt (×10^9^/L)	LDH (U/L)	Bil I (T) (μmol/L)	Haptoglobin (g/L)	Reticulocytes (×10^9^/L)	Coombs
Patient 1	112	92	306	575	31 (65)	<0.3	105.8	negative
Patient 2	67	84	234	361	25 (52)	<0.3	142	negative
Patient 3	114	94	349	285	26 (39)	0.08	110	negative
Patient 4	101	80	414	298	21 (27)	<0.3	230	negative

## Data Availability

The data presented in this study are available in this article.
